# Methyl-Containing Pharmaceuticals

**DOI:** 10.3390/ph17050563

**Published:** 2024-04-28

**Authors:** Davide Illuminati, Anna Fantinati

**Affiliations:** 1 Department of Life Sciences, University of Modena and Reggio Emilia, Via G. Campi, 213/d, 41125 Modena, Italy; 2 Department of Environmental and Prevention Sciences—DEPS, University of Ferrara, Via Fossato di Mortara 17, 44121 Ferrara, Italy

This Special Issue, which focused on methyl-containing pharmaceuticals, collected different papers and reviews on this topic [1,2,3]. The magic-methyl effect, well known in the field of medicinal chemistry and drug discovery, led to important implications that could be related not only to biological activity, selectivity, and affinity toward the target, but even to solubility, pharmacokinetic, and pharmacodynamic properties.

Indeed, the so called ‘magic-methyl effect’ has become relevant in the field of medicinal chemistry due to the huge pharmacological effects observed once the C-H bond was converted into a C-Me bond [4]. As reported above, the multiple implications related to this particular functionalization led to metabolic stability variations [5,6], pharmacodynamic/pharmacokinetic properties [7], and induced conformational effects [8,9], among plenty of other effects. 

Particularly, the marked implication of such an easy exchange has been well documented in the literature [10,11]; the conformational modification behavior could improve the adoption of bioactive conformation or disrupt spatial planarity and symmetry disposition, including increased lipophilicity [12].

Concerning this Special Issue, different aspects related with the methyl effect have been reported, including the selective redox-active, pro-oxidant, and pro-apoptotic properties of the methyl-containing selenylated imidazo[1,2-a]-pyridine derivative, which showed promising anticancer activity due to the activation of the antiproliferative pathway. [contribution1] Moreover, the multistep synthesis of novel bis-terephthalthioamides based on the methyl esters of amino acids was reported through an innovative microwave-assisted heating process. These compounds could potentially be employed as carriers through the generation of ADMET-friendly conglomerates. [contribution 2] Bello-Vargas et al. reported a computational method to improve the precision of predictive values, such as binding energies, interaction mode, and selectivity, for the interaction of small molecules comprising both methyl-containing and not methyl-containing compounds with COX isoforms. [contribution 3] The influence of methyl-containing non-natural amino acids was covered through a regio-selective synthesis of mono-methyl tyrosine (Mmt), exploited through the C(sp^2^)^_^H methylation reaction, in which a dibenzyl-moiety was used as a traceless bulky-regioselective inducer, allowing the 2-methyl tyrosine to be obtained alone. The non-natural amino acid was inserted into a peptide and compared to other derivatives through Ca^2+^ mobilization tests. [contribution 4] To conclude, this Special Issue was enriched by a review on this topic, which analyzed the relationship between chemical structures and biological activity, underlying the importance of the ‘magic-methyl effect’ and providing recently reported examples to acquire an overview of the current state of the art. Thus, this shed light on the methylation effect in bioactive compounds. [contribution 5].
